# Can *Anastatus bifasciatus* Be Used for Augmentative Biological Control of the Brown Marmorated Stink Bug in Fruit Orchards?

**DOI:** 10.3390/insects10040108

**Published:** 2019-04-15

**Authors:** Judith M. Stahl, Dirk Babendreier, Cristina Marazzi, Stefano Caruso, Elena Costi, Lara Maistrello, Tim Haye

**Affiliations:** 1 CABI, Rue des Grillons 1, 2800 Delémont, Switzerland; d.babendreier@cabi.org (D.B.); t.haye@cabi.org (T.H.); 2 Institute of Ecology and Evolutionary Biology, University of Bremen, Leobener Str. NW2, 28359 Bremen, Germany; 3 Servizio Fitosanitario Cantonale, Dipartimento Delle Finanze e Dell’economia, Sezione Dell’agricoltura Viale S. Franscini 17, 6501 Bellinzona, Switzerland; cristina.marazzi@ti.ch; 4 Consorzio Fitosanitario Provinciale di Modena, Via Santi Venceslao 14, 41123 Modena, Italy; stefano.caruso@regione.emilia-romagna.it; 5 Dipartimento di Scienze della Vita, Centro BIOGEST-SITEIA, Università di Modena e Reggio Emilia, Via G. Amendola 2, 42122 Reggio-Emilia, Italy; elena.costi@unimore.it (E.C.); lara.maistrello@unimore.it (L.M.)

**Keywords:** egg parasitoid, *Halyomorpha halys*, inundative release, invasive species, non-target effects, persistence

## Abstract

The generalist egg parasitoid *Anastatus bifasciatus* (Geoffroy) (Hymenoptera: Eupelmidae) is the most prevalent egg parasitoid of the invasive *Halyomorpha halys* (Stål) (Hemiptera: Pentatomidae) in Europe. To assess its efficacy against the pest *H. halys* and to validate the potential risks for non-target species in a realistic field setting, inundative releases were conducted over three consecutive years in four fruit orchards in Switzerland and Italy. In total, more than 4300 *A. bifasciatus* females were released, which was equivalent to 11,000 to 26,000 females per hectare, depending on distances between trees in each orchard. Parasitism of freeze-killed sentinel *H. halys* eggs achieved with the current release strategy was on average 6% (range: 2%–16%) and considered not high enough to effectively suppress the pest. However, the overall impact of *A. bifasciatus* on the mortality of *H. halys* eggs was likely underestimated. If pre-imaginal parasitoid mortality (3.3%) and host feeding (6%) are added to the observed parasitism (6%), the actual induced mortality of *H. halys* eggs may reach more than 15%. Parasitism of lepidopteran non-target species reached an average of 8% and thus, some degree of non-target parasitism after mass releases may be expected. To quantify the impact of the parasitoids in the orchards more precisely, naturally laid egg masses should be used in future trials to include host-finding cues of the host and host plants, and larger scale releases with potentially higher densities of parasitoids should be considered.

## 1. Introduction

Egg parasitoids (e.g., *Trichogramma* spp.) are advantageous for augmentative biological control because they reduce host populations before the damaging stages of the pest [1,2]. A less well-known group of parasitoids is the genus *Anastatus* Motschulsky (Hymenoptera: Eupelmidae), which comprises primary endoparasitoids of a wide variety of hosts in the insect orders Hemiptera, Lepidoptera, Blattodea, Orthoptera, and Mantodea [3,4,5]. *Anastatus* species are part of biological control programs worldwide and are used against a number of hemipteran pests such as fruitspotting bugs, *Amblypelta nitida* Stål and *A. lutescens lutescens* Distant (Coreidae), in Australian macadamia orchards [6,7,8], the citrus green stink bug, *Rhynchocoris humeralis* Thunberg (Pentatomidae), in Nepal [9] or the litchi stink bug, *Tessaratoma papillosa* Drury (Pentatomidae), in China [10,11,12]. In the Beijing Province of China, *Anastatus* sp. has been successfully mass released against the brown marmorated stink bug *Halyomorpha halys* (Stål) (Pentatomidae) with parasitism levels of more than 60% [13].

*Halyomorpha halys* is native to China, Japan as well as Korea, and has become invasive in the Americas and Europe in the mid-1990s and early 2000s, respectively [14,15,16]. Since its arrival in Switzerland, it has spread throughout many European countries [17]. It is a pest of a wide variety of economically important vegetable, fruit, and leguminous crops as well as ornamentals in both its native and invaded range [18,19]. Severe damage has been caused in fruit crops and hazelnuts in the USA, Georgia, and Italy [20,21,22]. In response, the number of insecticide treatments in certain regions has increased four-fold since the introduction of *H. halys,* thereby disrupting existing integrated pest management (IPM) programs, which led to outbreaks of secondary insect pests [20]. Due to the negative environmental effects of pesticide applications, environmentally friendly solutions such as biological control are needed. Augmentative biological control using native natural enemies against invasive species is a new approach and, to date, only a few examples exist, such as the use of *Trichopria drosophilae* Perkins (Hymenoptera: Diapriidae) against the spotted wing drosophila *Drosophila suzukii* (Matsumura) (Diptera: Drosophilidae) [23,24]. In Switzerland and Italy, *Anastatus bifasciatus* (Geoffroy) is the most prevalent native parasitoid successfully parasitizing *H. halys* eggs in the field [25,26,27]. It is one of the two European egg parasitoids capable of developing in viable *H. halys* eggs [26,28] and thus, it was selected as a potential candidate for inundative biological control of *H. halys* in Europe. Its host range, however, comprises more than 50 heteropteran and lepidopteran species and there are concerns that mass releases of *A. bifasciatus* might lead to undesired non-target effects [29,30].

The present experimental field study aimed to assess the efficacy of *A. bifasciatus* against *H. halys* and validate the potential risks for non-target species in a realistic field setting. Inundative releases were conducted over three consecutive years in four fruit orchards in Switzerland and Italy to develop a release strategy and answer the following questions: (1) What level of egg parasitism can be achieved by releasing *A. bifasciatus* against *H. halys* in fruit orchards? (2) Are non-target species parasitized by *A. bifasciatus* in the field? (3) Does *A. bifasciatus* persist in fruit orchards after releases?

## 2. Materials and Methods 

### 2.1. Parasitoid Rearing

The laboratory rearing of *A. bifasciatus* originated from two parasitized *H. halys* egg masses collected by S. Fischer (Agroscope Changins, Nyon, Switzerland) in the Canton of Valais, Switzerland, in 2014 [25]. Individuals of the founder population were identified by L. Fusu (University of Iasi, Romania). Approximately 50 adults (sex ratio 1:1) were kept in 100 × 115 mm mesh-top cylindrical plastic containers, placed above a 90 × 20 mm Petri dish filled with 1:10 honey water solution, which was provided to the parasitoids by two cotton wicks connecting the Petri dish to the plastic container. The rearing containers were stored in an incubator at a light/temperature cycle of L 16 h/20 °C and D 8 h/15 °C. Twice a week the wasps in each container were provided with approximately 150 new host eggs glued to cardboard pieces. The removed egg cards were stored at 26 °C until the emergence of the new generation. Newly emerged wasps were collected daily and transferred to the rearing containers. In the first year of the study (2016), *A. bifasciatus* was reared on eggs of *H. halys*. When females were seven days old, they were provided with new eggs until the day of release. In the second and third year (2017/2018), to increase the rearing and produce larger females [30] *A. bifasciatus* females were reared on a mix of *H. halys* and *Dendrolimus pini* (L.) (Lepidoptera: Lasiocampidae) eggs in Switzerland and on a mix of *H. halys* and *Manduca sexta* (Lepidoptera: Sphingidae) in Italy. *Halyomorpha halys* eggs were produced following the methods described in Reference [30].

### 2.2. Release Sites

Releases were conducted in three apple orchards in Switzerland and a single pear orchard in Italy (Table 1). In each orchard, an area of 60 trees in 4 neighboring rows (15 trees per row) located in the center of the orchard was selected as release plot. Depending on the distance between rows and between trees within rows the size of the plots varied between 210 and 480 m^2^ (Table 1). All sites were equipped with a data logger (“HOBO Pendant Temperature/Light 64K”, Onset Computer Corporation, Bourne, MA, USA), to record the ambient temperature for the length of the experiment.

### 2.3. Egg Exposure

#### 2.3.1. Parasitoid Efficacy and Persistence

*Halyomorpha halys* eggs were collected daily from the laboratory rearing, frozen for no longer than one month at −80 °C and thawed earliest two days before the release date. For exposure, egg masses with at least 20 eggs were used (mean ± SE: 26.0 ± 0.09). In each step of the experiment, sentinel *H. halys* egg masses were glued directly on the underside of leaves of apple/pear trees at a height of 50 to 180 cm. Branches of various host plants with feeding traces of *H. halys* were taken from the laboratory rearing cages and fixed next to the egg masses (one branch per egg mass) with a twist tie to increase the chance of parasitism by adding chemical cues of the host. Exposure times varied between four and seven days, depending on local weather conditions and phytosanitary treatment schedules (Table 2). 

**Figure 1:**
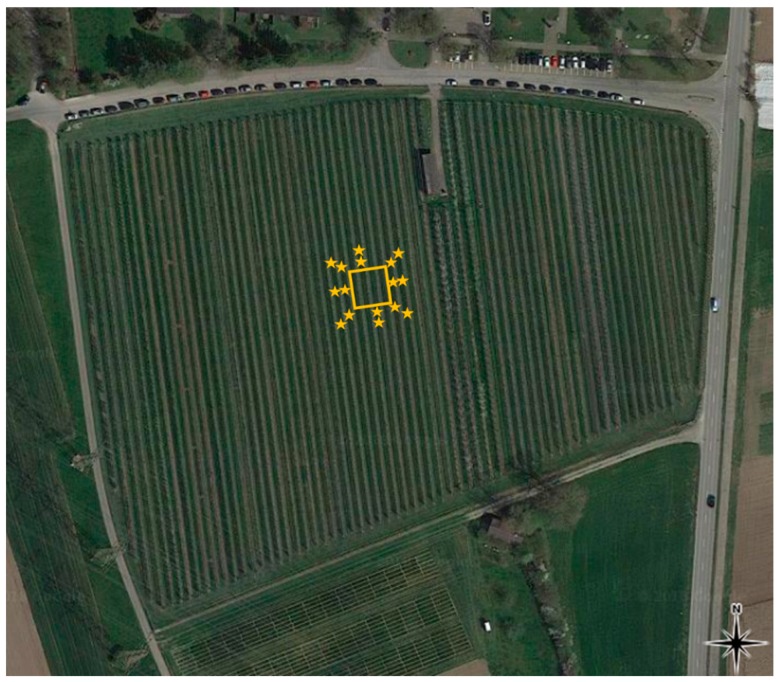
Site 1 with release plot (golden square) for releases 2016–2018, position of *H. halys* egg masses outside of release plot in 2016. Changed after Reference [31].

Five days prior releases, 25 *H. halys* egg masses were randomly distributed in each orchard, independent of its size, to measure parasitism of potential natural *A. bifasciatus* populations at each site (Table 2). On the day of the release, these egg masses were recollected, and 90 new egg masses were exposed inside the experimental plot (Table 2). Within the plot, 15 trees each were randomly equipped with 0, 1, 2, or 3 *H. halys* egg masses. During the first two releases in site 1 (2016), 32 *H. halys* egg masses each were exposed on 16 trees outside of, but in close proximity to, the experimental plot (up to eight meters distance) (Figure 1). Four to seven days after all parasitoid releases, exposed egg masses were recollected (Table 2). Fourteen days after the release, an additional 55 egg masses were placed out to monitor the persistence of the parasitoids (Table 2). Twenty-five of those egg masses were randomly distributed over 25 trees within the entire orchard, while the remaining 30 egg masses were placed on every second tree within the release plots.

#### 2.3.2. Non-target parasitism 

Depending on the availability of non-target species in each year of the study, eggs of six different lepidopteran species were exposed in the four release events (Table 3). In Switzerland, apart from *Euthrix potatoria* (L.) (Lasiocampidae), all non-target species were obtained from commercial insect breeders, the majority as pupae, and *Macrothylacia rubi* (L.) (Lasiocampidae) as eggs. *Euthrix potatoria* caterpillars were collected in Bärschwil, Switzerland, and reared until the adult stage in 50 × 50 × 50 cm gauze cages (“BugDorm-4090 Insect Rearing Cage”, MegaView Science Co. Ltd., Taichung, Taiwan) on *Dactylis glomerata* L. (Poaceae), which was replaced daily. Once the adult stage was reached, all non-target species were kept in a 50 × 50 × 50 cm gauze cage for oviposition and provided with honey water and if necessary, their associated host plants as oviposition stimulus. In Italy, adult females of *Macrothylacia rubi* (L.) (Lasiocampidae) and *Lasiocampa quercus* (L.) (Lasiocampidae) were collected in natural parks using light traps and placed in 50 × 50 × 50 cm gauze cages (one cage for each species) together with their host plants as oviposition stimulus. 

Newly laid eggs (<24 h) were frozen for no longer than one month at −80 °C and thawed no more than two days before the release date. Freeze-killed non-target egg batches of three to six eggs (see Table 3) were added to about half of the trees inside the release plot that were previously equipped with *H. halys* egg masses (28 and 24 out of 45 trees in 2016 and 2017/18, respectively). The density of non-target egg masses was matched with the densities of *H. halys* egg masses (0, 1, 2, or 3 egg masses), but not with the total number of eggs within the egg masses (Table 3). Non-target egg masses were randomly assigned to trees within the plot and egg batches were glued on the underside of leaves in close vicinity of the *H. halys* egg masses. 

In the first release event in 2016, mainly *E. potatoria* eggs were exposed, but to better understand the potential influence of different non-target hosts, seven additional trees within the plot were equipped with one *H. halys* and one *Samia cynthia* (Drury) (Saturniidae) egg mass each. In all following release events, only eggs of single non-target species were used, apart from the release at site 4, where each tree was equipped with three eggs of *M. rubi* and three eggs of *Lasiocampa quercus* (L.) (Lasiocampidae) (Table 3).

### 2.4. Parasitoid Releases 

All females used for releases were considered experienced with the target host because they were provided with *H. halys* eggs prior to releases. Females were deprived of hosts the week before release, so they would store their eggs and have a higher egg load when released. The day before release, females 1 to 5 weeks of age were transferred with glass pipettes into release devices and stored overnight under the rearing conditions described above. Release devices were made of clear plastic cups (8 cm high, 6 cm wide at the top). At the bottom, a small opening used for transferring the parasitoids was covered with a piece of foam dipped in honey to provide parasitoids with food. The top of the cup was covered with mesh wide enough (1.9 × 1.8 mm) to allow *A. bifasciatus* females to pass. For transport, the cups were closed with screw lids. In addition, cups were wrapped with black paper to ensure parasitoids would move upwards towards the natural light when cups were opened for release. Twelve cups containing 45 females each were equally distributed along the four rows of the plot (3/row) and placed at least five meters apart from each other. Cups were hung into the canopy at a height of 80 cm and fixed with twist ties. For release, screw lids were removed, and within the first hour, all parasitoids had left the containers. 

### 2.5. Treatment of Recollected Eggs

Recollected eggs were stored individually in small 54 × 14 mm Petri dishes at 26 °C, 70% RH, and a 16L:8D photoperiod. Eggs were counted and assigned to one of the following categories: collapsed, chewed, sucked (see Reference [32]) or intact eggs. Collapsed eggs were defined as eggs that looked undamaged but had lost more than half of their volume. 

Emerging wasps were counted, sexed and collected daily until no emergence was observed for four weeks. Parasitism was measured by the total number of eggs producing offspring divided by the sum of all intact eggs. Eggs that had been attacked by chewing or sucking predators or had collapsed in the field were excluded from the analysis since it was not possible to detect whether these eggs had been parasitized. Predation was calculated by the number of eggs showing signs of chewing or sucking divided by the sum of eggs with signs of predation and intact eggs. *Halyomorpha halys* and non-target egg masses recovered in 2017 were overwintered in 54 × 14 mm Petri dishes under outdoor conditions in an open wooden shelter [Canton Jura, Switzerland (N47°22’23; E 7°19’32)] four weeks after emergence had stopped. The following year, egg masses were checked daily for emergence from May onwards. 

### 2.6. Statistical Analyses

The influence of the distance to the closest release point on egg parasitism levels was analyzed for the first two years of the experiment, using a linear regression with the Theil-Sen estimator modified by Siegel repeated medians. The relationship between host density (measured as recovered *H. halys* eggs from a tree) and host impact was investigated using a generalized linear model (GLM) with a quasipoisson error distribution with the log link function. Statistical analyses were conducted with R version 3.2.3 [33] using the development environment RStudio Version 1.0.136 [34]. The package applied for the Theil-Sen estimator was ‘mblm’ [35].

## 3. Results

### 3.1. Parasitoid Efficacy and Persistence

Natural parasitism of sentinel egg masses prior to releases was overall low (site 2) or absent, with the exception of site 4 (Carpi), where it reached 28% (7 out of 25 egg masses). At site 1 (Lindau), parasitoids had been released in 2016, but no parasitism was detected in eggs exposed prior to releases in 2017.

**Figure 2:**
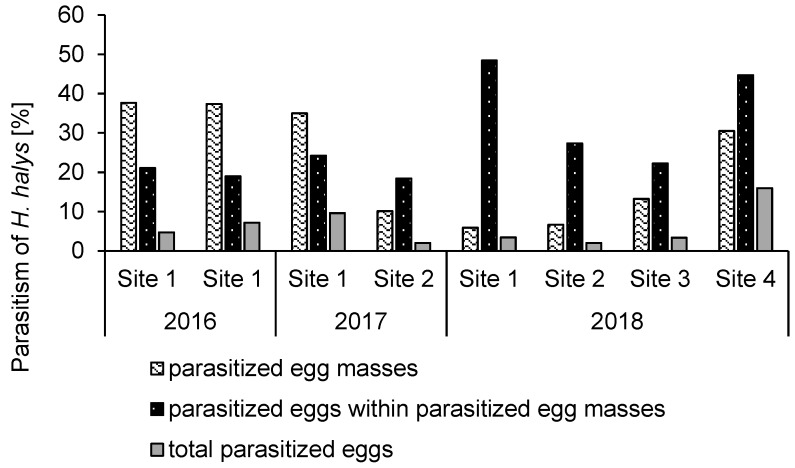
Parasitism measured by *A. bifasciatus* offspring emergence of sentinel *H. halys* egg masses exposed after *A. bifasciatus* releases for 4–7 days between 2016 and 2018 in Switzerland and Italy. Site numbers correspond with Table 1.

**Figure 3:**
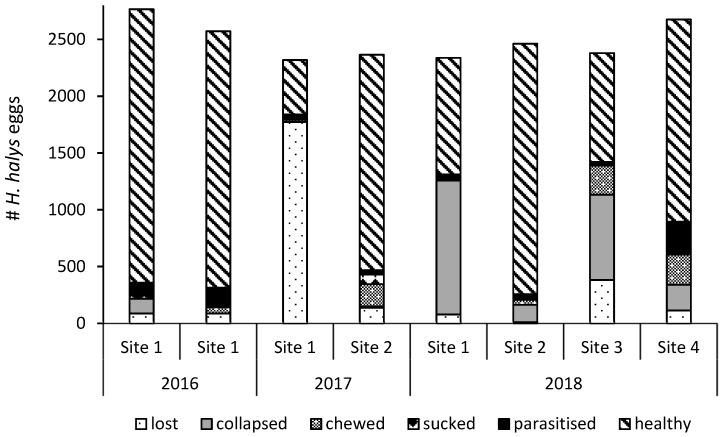
Fate of sentinel *H. halys* eggs exposed inside the release plots in Switzerland and Italy for 4–7 days after *A. bifasciatus* releases. Site numbers correspond with Table 1.

**Figure 4:**
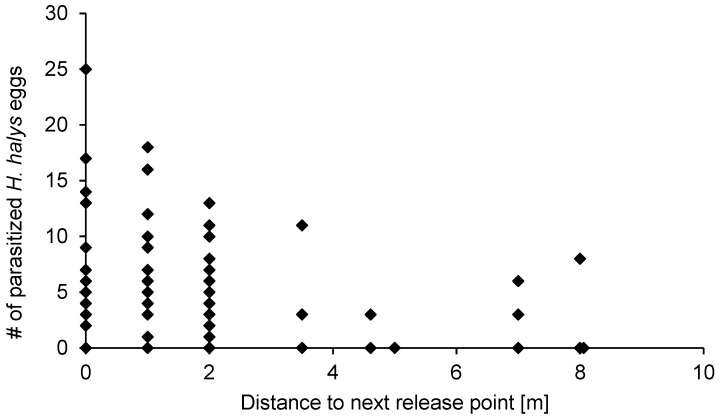
Number of *H. halys* eggs parasitized by *A. bifasciatus* depending on the distance to the closest release point during the first two releases in 2016 (site 1).

After releasing *A. bifasciatus*, parasitism of sentinel *H. halys* eggs was detected in each orchard (Figure 2). On average, 22.04% ± 5.41% (SE) (range: 6.67%–37.6%) (*n* = 8) of the recovered egg masses were parasitized. Since only an average of 28.2% ± 4.15% (SE) (range: 18.4%–48.4%) (*n* = 8) of the eggs in parasitized egg masses yielded parasitoid offspring (host exploitation), the actual egg parasitism was much lower, averaging 6.02% ± 1.70% (SE) (range: 2.00%–16.0%) (*n* = 8) when data from all years and release events were combined. Eggs that were exposed at sites 1 and 2 in July/August 2017 did not produce any *A. bifasciatus* offspring after overwintering in 2018. There was no significant relationship between host density, measured as the number of recovered eggs per tree, and number of parasitized eggs (quasipoisson GLM, df = 1,80, *χ*^2^ = 0.00775, *p* = 0.930). Both chewing and sucking predation was recorded at all sites (Figure 3), averaging 5.29% ± 1.83% (SE) (range: 0.04%–13.1%) and 0.789% ± 0.458% (SE) (range: 0.00%–3.88%) (both *n* = 8), respectively (Figure 3). *Halyomorpha halys* egg masses placed outside the release plots in the first two releases (site 1, 2016) yielded parasitoid offspring, but the level of parasitism was low. The number of parasitized *H. halys* eggs increased with decreasing distance to the nearest release point (linear regression, df = 119, *v* = 254.5, *p* < 0.001; Figure 4).

Sentinel *H. halys* egg masses exposed two weeks after *A. bifasciatus* releases yielded parasitoid offspring in three out of the eight release events. However, in the one case where natural *A. bifasciatus* population were found in the orchard, it remains unclear if detected parasitism was caused by released parasitoids or naturally occurring ones (site 4). On average, *A. bifasciatus* offspring emerged from 3.24% ± 1.73% (SE) (range: 0.00%–16.7%) (*n* = 9) of the recovered egg masses and from 2.30% ± 1.75% (SE) (range: 0.00%–16.0%) (*n* = 9) of the recovered eggs. 

### 3.2. Non-target Parasitism

**Figure 5:**
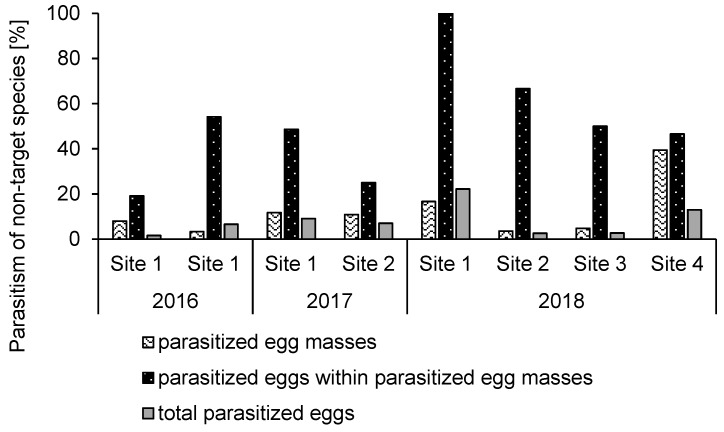
Parasitism measured by *A. bifasciatus* offspring emergence of sentinel non-target egg masses exposed after *A. bifasciatus* releases for 4–7 days between 2016 and 2018 in Switzerland and Italy. Site numbers correspond with Table 1.

Non-target parasitism occurred at all release sites, and five out of six non-target species yielded *A. bifasciatus* offspring. There was no indication of parasitism of *S. cynthia* eggs (recovered *n* = 29 eggs). The average parasitism of non-target eggs over all the eight releases was 8.11% ± 2.42% (SE) (*n* = 8), when eggs of the five parasitized species were combined (*E. potatoria:* 2.30%, *n* = 175; *O. pruni:* 6.70%, *n* = 120; *D. pini:* 7.58% *n* = 145; *L. quercus:* 11.7%, *n* = 103; *M. rubi:* 16.9%, *n* = 124) and ranged from 1.63% to 22.2% (*n* = 8) between release events (Figure 5). On average, 12.3% ± 4.20% (SE) (range: 3.3%1–39.4%) (*n* = 8) of the recovered non-target egg masses were parasitized, and an average of 51.3% ± 8.84% (SE) (range: 19.2%–100%) (*n* = 8) of the eggs in parasitized egg masses yielded parasitoid offspring (Figure 5).

## 4. Discussion

After experimental releases of *A. bifasciatus* females, moderate parasitism of sentinel *H. halys* eggs was detected in all eight release events at the four experimental sites. In two out of eight release events parasitism of sentinel eggs by natural *A. bifasciatus* populations was detected prior to releases. Accordingly, for these releases, it was impossible to distinguish if the observed parasitism following the releases was indeed caused by the released parasitoids or naturally occurring ones. In comparison, field releases of another European egg parasitoid, *Ooencyrtus telenomicida* (Vassiliev) (Hymenoptera: Encyrtidae) only elicited parasitism at 3 out of 6 release sites [26]. When estimating parasitism as the proportion of trees carrying at least one parasitized sentinel egg mass, *A. bifasciatus* was recovered from 30% of the trees. This is comparable with the results of experimental releases of *Anastatus* sp. in Australian macadamia orchards against fruitspotting bugs *Amblypelta nitida* Stål and *A. lutescens lutescens* Distant (Hemiptera: Coreidae), where parasitized host eggs were recovered from 24% of the plot trees [8]. 

Host exploitation (proportion of parasitized eggs within an egg mass) by *A. bifasciatus* was rather low (28%), which may be explained by the low weekly fecundity of the parasitoids [36], assuming that parasitized egg masses were visited by single or few females. In the present study, *A. bifasciatus* females were deprived of host eggs for one-week prior releases to increase the number of stored eggs and parasitoids’ host searching motivation [37], but this approach did not result in higher host exploitation. Unfortunately, there is no information about the behavior of *A. bifasciatus* in the field that might help explain the low exploitation values and improve release efficacy. To increase host exploitation by decreasing patch-leaving stimuli, dispersal capacities can be reduced by arresting natural enemies within the patch with chemical cues [37]. 

When data from all releases were combined, total egg parasitism was on average 6%. Based on the mean host exploitation value in the field (6.70 eggs), only 1% of the 540 released females would have been responsible for the observed parasitism if each of the egg masses was found by only one female. This could also be connected to the small experimental plots, where dispersal is more likely than from bigger areas such as a whole orchard. Calculations carried out by Reference [38] after releases of *Trichogramma nubilale* Ertle and Davis (Hymenoptera: Trichogrammatidae) revealed that every day 40% of the parasitoid females disappeared, which was attributed to the hot and dry weather conditions. In the present study, weather conditions at release sites were extremely hot and dry over the three years of the study and may have caused some degree of adult mortality. Another factor that might have influenced observed parasitism levels is intraguild predation. Some sentinel eggs that showed signs of predation also had *A. bifasciatus* host feeding marks, indicating that at least some parasitized eggs had been eaten by predators. As interactions between parasitoids and predators of *H. halys* have not been studied so far [28], future studies on the subject could provide valuable input. 

Host location of parasitoids is facilitated by a number of cues that can either be emitted by the host plant [39], the host itself [40] or a combination of the two factors. The experimental design of the current study was aimed to provide natural conditions and include host cues provided by branches with feeding and walking traces of *H. halys* next to the sentinel egg masses, assuming that these traces guide parasitoids to their hosts, as demonstrated for *Trissolcus basalis* (Wollaston) (Hymenoptera: Scelionidae) locating egg masses of *Nezara viridula* (L.) (Heteroptera: Pentatomidae) [41]. At the beginning of the release experiments, the factors influencing the host finding of *A. bifasciatus* were unknown, but in the meantime [42] demonstrated that *A. bifasciatus* positively responded to adult *H. halys* male volatiles and to *H. halys*-induced plant volatiles, indicating ability to exploit cues associated with the new host for egg location. Consequently, sentinel *H. halys* egg masses exposed in this study may have lacked important chemical cues associated with *H. halys* egg masses in nature, resulting in much lower host finding and thus, lower parasitism by *A. bifasciatus*. In a preliminary trial at site 4, reproductive *H. halys* adults were set up in sleeve cages on randomly selected trees two days before the *A. bifasciatus* release with the aim to expose naturally laid and frozen egg masses simultaneously. Although the number of egg masses laid in the sleeve cages (*n* = 8) was too low to obtain meaningful results, parasitism of naturally laid eggs (*n* = 99) was remarkably higher (48.5%) compared to the frozen sentinel eggs (16%), indicating the importance of considering naturally laid egg masses in future release trials (LM, EC, SC, unpublished data).

Apart from trying to increase host exploitation, releasing higher numbers of parasitoids is another alternative to increase pest suppression. In the present study, the release density was 540 female *A. bifasciatus* per 60 trees, which translates to 11,000 (site 4) to 26,000 (site 1) females per hectare, depending on the distances between rows and trees within rows. In comparison, many commercial and experimental releases involve larger quantities of parasitoids, such as several times 100,000 *Trichogramma* per hectare [43]. In China, commercial releases of the closely related *A. japonicus* against less severe infestations of the litchi stink bug, *T. papillosa,* required180 females per medium-sized tree, which is significantly more than in the present experiments, and yield an average of 52–94% parasitism in the first year of its releases [44]. 

Measuring parasitism by offspring emergence is less labor-intensive than dissections, but underestimates the actual levels of parasitism if a proportion of individuals cannot undergo complete development. Dissections of parasitized hosts often have the disadvantage that tiny eggs and early instar larvae of parasitoids can be hard to detect inside hosts, and remains of dead parasitoids may be difficult to recognize when hosts decay. An alternative method to detect parasitoids inside their hosts is the use of molecular markers [45,46]. The analysis of remaining unemerged eggs from parasitized egg masses from site 1 in 2017 showed that 14% contained dead developmental stages of *A. bifasciatus* [47], suggesting that host exploitation was indeed much higher than what was measured by offspring emergence. Another important behavioral trait of *A. bifasciatus* is host feeding [36]. Since many parasitoids kill hosts by host feeding as well as parasitism, this is a factor that should not be neglected when estimating the efficacy of a parasitoid. To avoid additional damage by increasing *H. halys* densities in the fruit orchards, freeze-killed *H. halys* egg masses had to be used for the experiments. As a consequence, it was not possible to assess the number of host eggs killed by *A. bifasciatus* host feeding in the field. However, data from previous laboratory studies suggest that the number of eggs killed by host feeding is nearly as high as the number of eggs killed by parasitization, which may double the estimated host mortality [36,48]. In addition, parasitoid efficacy might be distinctly higher in a real infestation situation because using sentinel egg masses may underestimate parasitism [49]. If preimaginal parasitoid mortality (3.3%) and host feeding (6%) are added to the observed parasitism (6% offspring emergence), the actual induced mortality of *H. halys* eggs may have been 15.3%.

Higher parasitism of *H. halys* may be accompanied by higher non-target parasitism since the host impact values for non-targets (8%) and the target (6%) are similar. These findings agree with studies on the physiological host range of *A. bifasciatus,* showing that most non-target species where as frequently parasitized and suitable for development as the target host [36]. Even though arthropod biodiversity in apple orchards tends to be higher than in annual crops [50,51], reviewed by [52], insect diversity, in other words, the number of potential non-targets, was still comparably low at our experimental sites. Consequently, potential dispersal of released *A. bifasciatus* into habitats outside the orchards is a more important factor in its risk assessment [53]. In this study, the parasitoid movement could only be confirmed up to eight meters from the closest release point. Other *Anastatus* species, however, can disperse up to 60 meters [8,44] and, with wind dispersal, up to 100 meters [44]. Since parasitism levels of sentinel eggs were low, the results of the experiment looking at parasitoid movement are not conclusive and further investigations are needed. As *A. bifasciatus* has the potential longevity of three months (97.5 days when provided with honey water) [36], released wasps were expected to persist in the fruit orchards, but their presence was only retained for two weeks in three out of eight release events which could also be attributed to dispersal. Since releases were conducted in late summer, nectar sources were hardly available in the orchards, which may have caused a large proportion of parasitoids to leave the orchards and disperse into other habitats.

## 5. Conclusions

Field releases of *A. bifasciatus* can increase parasitism of *H. halys* eggs in fruit orchards, but parasitism levels achieved with the current release strategy were not high enough to effectively suppress the pest. However, the overall impact is likely higher when mortality of parasitoid eggs and larvae inside host eggs and host egg mortality by host feeding are taken into account. Consequently, releasing higher densities of parasitoids, and at a larger scale to reduce the impact of dispersal, should be considered. In addition, naturally laid egg masses should be used in future trials to include host-finding cues of the host and host plants and avoid potential adverse effects of frozen sentinel eggs. If those changes result in an increase of overall parasitism under field conditions, future augmentative releases should be carried out in correspondence with the egg laying peak of the overwintered generation in May/June to have a greater impact in reducing *H. halys* populations along the entire season. Some degree of non-target parasitism after mass releases can be expected, but whether non-targets would be negatively affected at the population level will require further investigations, including dispersal studies. 

## Figures and Tables

**Table 1:** Field sites for experimental *A. bifasciatus* releases between 2016 and 2018.

Site	Location (Municipality, Canton/Region, Country)	GPS Coordinates	Management	Host Plants in Plot (Species, Variety)	*H. halys* Presence	Release Plot Size [m^2^]	Number of Releases
1	Lindau, Zurich, Switzerland	47°26’52.0″N 8°40’47.6″E	IPM	*Malus pumila*, Golden Delicious/Diwa/Braeburn	no	210	4
2	Bellinzona, Ticino, Switzerland	46°09’42.1″N 8°58’12.2″E	IPM	*Malus pumila*, Golden Delicious/Braeburn	yes	288	2
3	Manno, Ticino, Switzerland	46°01’52.8″N 8°55’20.4″E	Organic	*Malus pumila*, unknown varieties	yes	212	1
4	Carpi, Emilia-Romagna, Italy	44°43’46.8″N 10°52’30.0″E	Organic	*Pyrus communis,* Abate Fetel	yes	480	1

**Table 2:** Overview of *H. halys* egg masses exposed before parasitoid releases (“pre-release monitoring”), directly after releases within the release plot (“release”) and two weeks after releases (“post-release monitoring”).

Exposure Date	Location	Treatment	Mean Temperature (Min – Max) [°C]	Egg Masses (Eggs) Exposed	Egg Masses (Eggs) Recovered ^1^
**2016**					
23.–28. Jul	1 – Lindau	Pre-release monitoring	22.4 (12.9–36.0)	25 (657)	22 (575)
28. Jul – 2. Aug		Release	21.7 (12.4–38.3)	97 (2653)	93 (2409)
11.–16. Aug		Post-release monitoring	21.8 (9.6–36.6)	55 (1480)	54 (1459)
16.–21. Aug		Release	20.4 (12.1–36.6)	90 (2410)	83 (2258)
**2017**					
17.–21. Jul	1 – Lindau	Pre-release monitoring	23.0 (13.8–34.5)	25 (623)	15 (298)
29. Jul–3. Aug		Release	23.2 (15.3–35.0)	90 (2273)	20 (479)
10.–17. Aug		Post-release monitoring	18.8 (10.2–32.7)	55 (1410)	23 (562)
2.–10. Aug	2 – Bellinzona	Pre-release monitoring	23.4 (15.3–38.5)	25 (637)	20 (416)
14.–21. Aug		Release	23.1 (10.9–35.2)	90 (2327)	89 (1897)
28. Aug–4. Sep		Post-release monitoring	21.2 (12.3–33.5)	55 (1387)	53 (774)
**2018**					
19.–25. Jul	2 – Bellinzona	Pre-release monitoring	25.0 (13.9–38.3)	25 (685)	22 (1371)
25.–30. Jul		Release	26.1 (15.5–37.9)	90 (2418)	90 (2205)
8.–13. Aug		Post-release monitoring	23.7 (16.8–36.4)	55 (2697)	51 (1940)
19.–25. Jul	4 – Carpi	Pre-release monitoring	25.0 (16.0–35.0)	25 (694)	25 (363)
25.–30. Jul		Release	26.5 (17.0–36.5)	90 (2392)	82 (1780)
9.–14. Aug		Post-release monitoring	25.4 (17.0–35.5)	55 (1469)	55 (958)
25.–30. Jul	3 – Manno	Pre-release monitoring	26.6 (15.3–44.5)	25 (752)	20 (545)
30. Jul–3. Aug		Release	28.0 (17.5–39.4)	90 (2347)	68 (957)
13.–17. Aug		Post-release monitoring	23.1 (14.6–34.7)	55 (1677)	51 (1345)
6.–10. Aug	1 – Lindau	Pre-release monitoring	Na	25 (611)	10 (58)
11.–15. Aug		Release	Na	90 (2303)	85 (1026)
24.–28. Aug		Post-release monitoring	Na	55 (1760)	51 (1536)
**Total**				**1287 (35,662)**	**1082 (25,340)**

^1^ healthy looking eggs, neither collapsed nor predated on.

**Table 3:** Exposure of non-target eggs during experimental *A. bifasciatus* field releases 2016–2018.

Species	Site	Year	Egg Batch Size	Total # Eggs Exposed/Site
*Samia cynthia* (Drury) (Saturniidae)	1	2016	6	42
*Euthrix potatoria* (L.) (Lasiocampidae)	1	2016	6	252
*Odonestis pruni* (L.) (Lasiocampidae)	1	2016	4	168
*Dendrolimus pini* (L.) (Lasiocampidae)	1, 2	2017	4	192
*Lasiocampa quercus* (L.) (Lasiocampidae)	1, 2, 3, 4	2018	6 (3 in site 4)	288 (144)
*Macrothylacia rubi* (L.) (Lasiocampidae)	4	2018	3	144
